# Prognostic classification in acute exacerbation of idiopathic pulmonary fibrosis: a multicentre retrospective cohort study

**DOI:** 10.1038/s41598-021-88718-2

**Published:** 2021-04-27

**Authors:** Takahito Suzuki, Hironao Hozumi, Koichi Miyashita, Masato Kono, Yuzo Suzuki, Masato Karayama, Kazuki Furuhashi, Hirotsugu Hasegawa, Tomoyuki Fujisawa, Noriyuki Enomoto, Yutaro Nakamura, Naoki Inui, Koshi Yokomura, Hidenori Nakamura, Takafumi Suda

**Affiliations:** 1 Second Division, Department of Internal Medicine, Hamamatsu University School of Medicine, 1-20-1 Handayama Higashiku, Hamamatsu, 431-3192 Japan; 2 Department of Respiratory Medicine, Seirei Hamamatsu General Hospital, Hamamatsu, Japan; 3 Department of Respiratory Medicine, Seirei Mikatahara General Hospital, Hamamatsu, Japan; 4 Department of Clinical Pharmacology and Therapeutics, Hamamatsu University School of Medicine, 1-20-1 Handayama Higashiku, Hamamatsu, 431-3192 Japan

**Keywords:** Respiratory tract diseases, Medical research

## Abstract

Acute exacerbation (AE) in idiopathic pulmonary fibrosis (IPF) is a major prognostic determinant. However, evidence for its prognostic strength is mainly based on the results of small cohort studies with statistical limitations. This retrospective study, which included 108 patients with a first episode of AE-IPF, aimed to identify prognostic factors and to develop prognostic classification models. Multivariate Cox regression analysis revealed that a lower percent-predicted forced vital capacity within 12 months before AE onset (baseline %FVC) and a lower PaO_2_/FiO_2_ ratio at AE onset were independent mortality predictors. If the value of each predictor was lower than the cutoff determined by receiver-operating characteristic analysis, 1 point was assigned. Classification of patients into mild, moderate, and severe groups based on total score showed post-AE 90-day cumulative survival rates of 83.3%, 66.2%, and 22.2%, respectively (model 1: C-index 0.702). Moreover, a decision tree-based model was created with the recursive partitioning method using baseline %FVC and PaO_2_/FiO_2_ ratio at AE onset from among multivariable; accordingly, patients were classified into 3 groups with post-AE 90-day cumulative survival rates of 84.1%, 64.3%, and 24.0%, respectively (model 2: C-index 0.735). These models can guide clinicians in determining therapeutic strategies and help design future studies on AE-IPF.

## Introduction

Idiopathic pulmonary fibrosis (IPF) is a chronic progressive fibrotic lung disease of unknown aetiology with a median survival time of 2–4 years from diagnosis^1,2^. Acute exacerbation (AE) is a rapidly progressive and fatal deterioration in respiratory function that unpredictably develops in IPF patients^3^. The annual incidence of AE-IPF is 5–15%, and the 90-day mortality rate is very high, at 30–60%^3^. To improve the survival rate for such a fatal condition, establishment of management and treatment strategies is urgently needed.

The AE-IPF definition was revised in 2016, which improved diagnostic feasibility of the criteria for clinicians and investigators^3,4^. However, these diagnostic criteria do not define the disease severity. Due to the broader definition of AE-IPF, it is likely that the diagnosed patients have a wide range of disease severity and varied clinical course^5^. Differences in severity and estimated prognosis may affect the treatment strategy for patients and clinical trial design. The already-reported prognostic factors in AE-IPF patients are mainly based on the results of relatively small studies with statistical limitations and have not yet been fully validated and therefore need more investigation^3,6–10^. In addition, to date, no prognostic classification has been established for AE-IPF. In acute respiratory distress syndrome (ARDS), a severity classification has been proposed based on the arterial oxygen partial pressure (PaO_2_)/fractional inspired oxygen (FiO_2_) ratio (P/F), which has influenced real-world clinical practice and study design^11^. Likewise, for AE-IPF patients, indicators, such as prognostic factors/classification, would provide helpful information to attending physicians, patients and families, and researchers. This study aimed to identify clinical and physiologic factors associated with prognosis in a large cohort of AE-IPF patients and to develop a simple-to-use prognostic classification model based on the identified prognostic factors.

## Methods

### Participants

**Figure 1:**
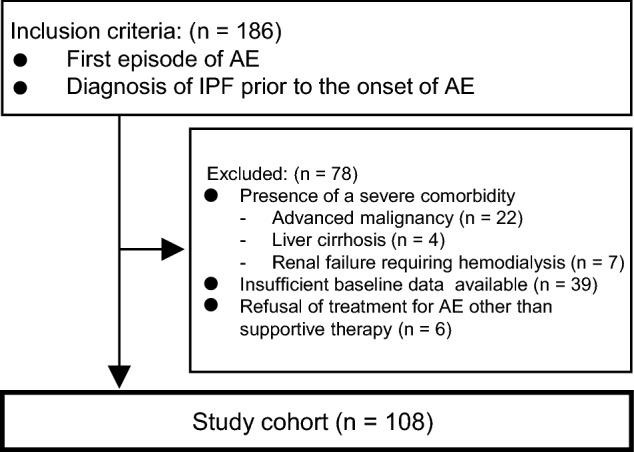
Flow diagram of patient selection. *AE* acute exacerbation, *IPF* idiopathic pulmonary fibrosis.

This retrospective review assessed 186 consecutive patients with a first episode of AE-IPF diagnosed between 2004 and 2017 at the Hamamatsu University, Seirei Mikatahara General, or Seirei Hamamatsu General Hospitals in Japan. The diagnosis of IPF was based on the international guideline^1^.[1, 6] All patients with AE-IPF were diagnosed with IPF before AE onset. The 2016 International Working Group report was used as the basis for AE-IPF diagnosis^3^. Both the diagnoses of IPF and AE were reassessed for this study by the investigators and determined on the basis of their consensus. Figure 1 shows a flow diagram of patient selection. Exclusion criteria were: presence of severe comorbidity at diagnosis (advanced malignancy, liver cirrhosis, renal failure requiring hemodialysis); insufficient baseline data before AE onset (e.g., percent-predicted forced vital capacity [%FVC]); and patient refusal of treatment for AE other than supportive therapy, which excluded 78 patients. Consequently, the study enrolled 108 patients with a first episode of AE-IPF. This multicentre study was conducted in accordance with the Declaration of Helsinki. The institutional review board of Hamamatsu University School of Medicine, the institutional review board of Seirei Mikatahara General Hospital, and the institutional review board of Seirei Hamamatsu General Hospital approved this study (Hamamatsu University School of Medicine, Approval Number 18-023; Seirei Mikatahara General Hospital, Approval Number 18-23; Seirei Hamamatsu General Hospital, Approval Number 2750) and waived the need for written informed consents because of the retrospective nature of the study.

### Data collection

These data were collected retrospectively from medical records: data before AE onset, including age at IPF diagnosis, sex, smoking history, %FVC and percent-predicted diffusing capacity of the lung carbon monoxide within 12 months before AE onset (baseline %FVC and baseline %DL_CO_, respectively), Gender–Age–Physiology (GAP) stage^12^, high-resolution computed tomography (HRCT), and treatment for IPF, data at the time of AE onset (prior to treatment for AE, including mechanical ventilation), including age, P/F ratio, treatment for AE-IPF, and outcomes. Relapse-free survival for AE was defined as the time from diagnosis of the first AE until the AE relapse date or until the last visit (the censoring date or mortality date) for AE patients who did not relapse. Post-AE survival was defined as the time from diagnosis of the first AE until the last visit. Patients were censored if they remained alive until August 31, 2019.

### Statistical analysis

All values were expressed as median (interquartile range [IQR]) or number (%). Post-AE cumulative survival and AE relapse rates were evaluated using the Kaplan–Meier method; between-group survival differences were assessed using the Wilcoxon test. To identify prognostic factors, Cox regression analysis (with/without time-dependent covariates) was used; thereafter, hazard ratio (HR), 95% confidence interval (CI), and* p* values were calculated. Age, sex, already-reported physiologic prognostic factors of IPF (baseline %FVC and baseline %DL_CO_)^12^, and all variables that were statistically significant in univariate analyses were included for multivariate analyses. Prognostic classification model 1 was generated on the basis of the prognostic factors identified. Briefly, 1 point was assigned to each prognostic factor and patients were categorized into groups based on the sum of these scores. Receiver-operating characteristic curve analysis was used to determine an optimal cutoff value of continuous variables (Youden index). Prognostic classification model 2 was generated on the basis of recursive partitioning creating a decision tree. Its node splitting was based on the LogWorth statistics [− log_10_ (*p* value)], and the candidate variable for the split that maximizes LogWorth was determined to be the optimal split, which was reported in candidate reports. The discrimination performance of the model was evaluated using the concordance statistic (C-index). Subdistribution hazard analyses were performed using the Fine and Gray method to identify the predictors of AE relapse (treating mortality as a competing event); the already-reported risk factors of AE development (baseline %FVC and baseline %DL_CO_)^3,6^ and all variables that were statistically significant in univariate analyses were included for multivariate analyses. In all analyses, *p* < 0.05 was considered statistically significant. The Holm method was used to adjust *p* values in multiple comparisons. Statistical analyses were performed using the R software version 4.0.2 (The R Foundation for Statistical Computing, Vienna, Austria) and JMP software version 13.2.1 (SAS Institute Inc., NC, USA).

## Results

### Characteristics, treatment, and outcome

Table 1 shows the characteristics of 108 patients with AE-IPF. Median age at diagnosis of IPF was 72 years; 93 (86.1%) patients were male. Median baseline % FVC and baseline % DL_CO_ were 68.5% and 61.1%, respectively. Median time from IPF diagnosis to first AE onset was 23.4 months (IQR, 6.0–45.6 month). Median age at first AE-IPF was 74 years. Median P/F ratio at AE onset was 223 Torr. For first-line treatment for AE-IPF, all patients were treated with corticosteroid therapy (with/without an immunosuppressant).

**Table 1:** Characteristics of the AE-IPF cohort.

	n = 108
**Before AE**	
Age at IPF diagnosis, years	72 (66–77)
Gender, male	93 (86.1)
Smoking, current or ex	91 (84.3)
IPF diagnosis, clinical/pathologically proven	83 (76.9)/25 (23.2)
% FVC within 12 months before first AE, %	68.5 (54.2–85.7)
% DL_CO_ within 12 months before first AE, %	61.1 (20–124)
GAP stage, I/II/III	50 (46.3)/48 (44.4)/10 (9.3)
HRCT pattern within 12 months before first AE, UIP	95 (88.0)
Treatment for IPF prior to AE	
Anti-inflammatory	18 (16.7)
CS/CS + oral CY/CS + CyA	11 (10.2)/3 (2.8)/4 (3.7)
Antifibrotic	20 (18.5)
Pirfenidone/Nintedanib	15 (13.9)/5 (4.6)
**At AE onset**	
Age, years	74 (69–79)
PaO_2_/FiO_2_ ratio, Torr	223 (167–276)
KL-6, U/mL	1530 (973–2540)
SP-D, ng/mL	323 (202–505)
**Treatment for first AE**	
Anti-inflammatory, CS/CS + IVCY/CS + CyA	67 (62.0)/38 (35.2)/3 (2.8)
Polymyxin-B direct hemoperfusion	26 (24.1)
**AE relapse, yes**	27 (25)
AE relapse-free survival, day	148 (31–443)
**Post-AE survival, day**	238 (31–547)
**Death during study period**	94 (87.0)
Death cause, respiratory-related/infection/others	78 (83.0)/8 (8.5)/8 (8.5)

Data are presented as n (%) or median (interquartile range).

*IPF* idiopathic pulmonary fibrosis, *FVC* forced vital capacity, *DL*_*CO*_ diffusing capacity of the lung carbon monoxide, *GAP* gender–age–physiology index, *AE* acute exacerbation, *HRCT* high-resolution computed tomography, *UIP* usual interstitial pneumonia, *CS* corticosteroids, *CY* cyclophosphamide, *CyA* cyclosporin A, *IVCY* intravenous CY, *KL-6* Krebs von den Lungen-6, *SP-D* surfactant protein D.

**Figure 2:**
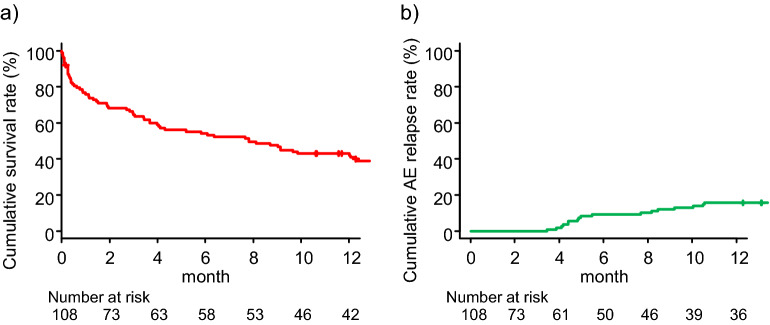
Post-AE cumulative survival and AE relapse rates. (**a**) The post-AE 90-day and 1-year cumulative survival rates were 63.9% (95% confidence interval [CI] 55.7–73.6) and 42.6% (95% CI 32.6–51.1), respectively, and (**b**) 1-year cumulative AE relapse rate was 19.0% (95% CI 12.2–27.4). *AE* acute exacerbation, *IPF* idiopathic pulmonary fibrosis.

During the post-AE period, 27 (25%) patients experienced AE relapse and 94 (87%) died. The major cause of mortality was respiratory-related conditions, including AE (57 patients), chronic respiratory failure after AE (20 patients), and pneumothorax (1 patient), followed by infection (8 patients), cardiovascular event (4 patients), lung cancer (1 patient), and unknown causes (3 patients). The post-AE 90-day and 1-year cumulative survival rates were 63.9% and 42.6%, respectively (Fig. 2a). The 1-year cumulative AE relapse rate was 19.0% (Fig. 2b).

### Prognostic factors for AE-IPF

Table 2 shows the results of Cox proportional hazard analysis for mortality after AE onset. In the univariate analysis, anti-inflammatory treatment for IPF before first AE and P/F ratio at AE onset were associated with mortality after AE onset. On multivariate analysis, lower baseline %FVC and lower P/F ratio at AE onset were independent prognostic factors. Even in multivariate analyses adjusted for AE treatment, lower baseline %FVC and lower P/F ratio at AE onset were independently associated with a shorter survival (Supplementary Table S1).

**Table 2:** Results of cox hazards analysis of mortality.

	HR	95% CI	*P*-value
Univariate model			
Male (vs. female)	0.96	0.56–1.77	0.88
Smoking, current or ex (vs. never)	0.82	0.49–1.46	0.48
Baseline % FVC^a^, per 1% increase	0.99	0.98–1.00	0.14
Baseline % DL_CO_^a^, per 1% increase	0.99	0.98–1.00	0.18
Baseline GAP stage^a^			
II (vs. I)	0.86	0.55–1.33	0.50
III (vs. II)	2.13	0.99–4.16	0.06
UIP pattern on HRCT^a^ (vs. other patterns)	1.27	0.65–2.45	0.48
Treatment for IPF prior to first AE			
Anti-inflammatory, yes (vs. no)	2.01	1.14–3.35	0.02
Antifibrotic, yes (vs. no)	1.13	0.65–1.85	0.66
Pirfenidone	0.98	0.53–1.69	0.95
Nintedanib	1.90	0.58–4.62	0.26
At AE onset			
Age, years	0.99	0.97–1.02	0.59
PaO_2_/FiO_2_ ratio, per 10 Torr increase	0.96	0.93–0.99	< 0.01
KL-6, per 100 U/mL increase	0.99	0.97–1.01	0.44
SP-D, per 100 ng/mL increase	0.97	0.90–1.03	0.32
Treatment for first AE			
CS + IS (vs. CS)	1.01	0.66–1.52	0.97
Polymyxin-B direct hemoperfusion, yes (vs. no)	1.01	0.62–1.60	0.95
Multivariate model			
Age at AE onset, years	1.02	0.98–1.06	0.32
Male (vs. female)	0.74	0.31–2.07	0.54
Baseline % FVC^a^, per 1% increase	0.97	0.95–0.99	< 0.01
Baseline % DL_CO_^a^, per 1% increase	1.01	0.99–1.03	0.15
Anti-inflammatory treatment prior to AE, yes (vs. no)	1.88	0.84–3.77	0.12
PaO_2_/FiO_2_ ratio at AE onset, per 10 Torr increase	0.89	0.84–0.95	< 0.01

*HR* hazards ratio, *CI* confidence interval, *AE* acute exacerbation, *FVC* forced vital capacity, *DL*_*CO*_ diffusing capacity of the lung carbon monoxide, *GAP* gender–age–physiology index, *UIP* usual interstitial pneumonia, *HRCT* high-resolution computed tomography, *CS* corticosteroids, *IS* immunosuppressant, *PMX-DHP* polymyxin-B direct hemoperfusion, *KL-6* Krebs von den Lungen-6, *SP-D* surfactant protein D.

^a^Within 12 months before first AE.

### Prognostic classification model 1

**Figure 3:**
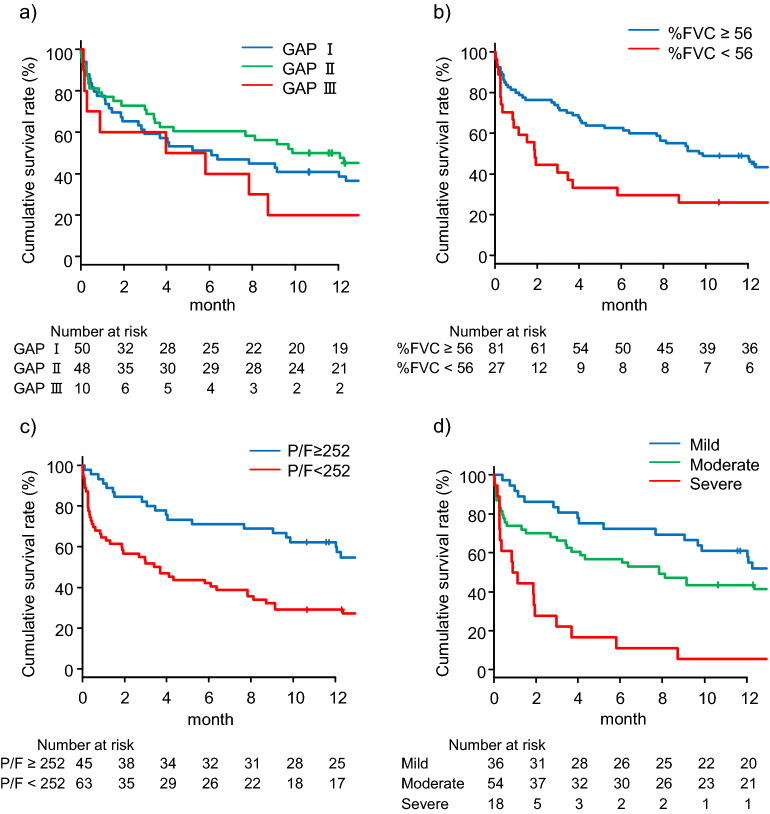
Post-AE cumulative survival rates by the GAP model and prognostic classification model 1. (**a**) The post-AE 90-day cumulative survival rates of the patients by GAP stage: GAP stage I, 59.3% (95% CI 44.3–71.5); GAP stage II, 68.8% (95% CI 53.6–79.8); and GAP stage III, 60% (95% CI 25.3–82.7), with GAP stage I compared with GAP stage II (adjusted *p* = 0.45 by Wilcoxon test) and GAP stage II compared with GAP stage III (adjusted *p* = 0.26, by Wilcoxon test). The discrimination performance (C-index) of this model for post-AE 90-day survival was 0.526 (95% CI 0.417–0.634). (**b**) The post-AE 90-day cumulative survival rates of the patients with AE-IPF who had %FVCs ≥ 56% and those with %FVCs < 56% were 72.6% (95% CI 61.4–81.0) and 40.7% (95% CI 22.5–58.2), respectively (*p* < 0.01 by Wilcoxon test). (**c**) The post-AE 90-day cumulative survival rates of the patients with AE-IPF who had P/F ratios ≥ 252 Torr and those with P/F ratios < 252 Torr were 82.2% (95% CI 67.6–90.7) and 50.8% (95% CI 38.7–63.3), respectively (*p* < 0.01 by Wilcoxon test). (**d**) The post-AE 90-day cumulative survival rates of the patients by total risk scores of 0 (mild), 1 (moderate), and 2 (severe) were 83.3% (95% CI 66.7–92.1), 66.2% (95% CI 51.9–77.2), and 22.2% (95% CI 6.9–42.9), with the mild group compared with the moderate group (adjusted *p* = 0.04 by Wilcoxon test) and the moderate group compared with the severe group (adjusted *p* < 0.01 by Wilcoxon test). The discrimination performance (C-index) of this model for post-AE 90-day survival was 0.702 (95% CI 0.61–0.80). *AE* acute exacerbation, *FVC* forced Vital Capacity, *GAP* Gender-Age-Physiology, *IPF* idiopathic pulmonary fibrosis, *P/F* PaO_2_-to-FiO_2_ ratio.

Figure 3a shows survival curves for patients with AE-IPF by baseline GAP stage. The post-AE 90-day cumulative survival rates of patients with GAP stages I, II, and III were 59.3%, 68.8%, and 60%, respectively. No significant differences were noted in adjacent survival curves (GAP stage I vs. II, adjusted *p* = 0.45; GAP stage II vs. III, adjusted *p* = 0.26). The discrimination performance (C-index) of this model for mortality within 90 days of AE onset was 0.526.

Next, the baseline %FVC and P/F ratio at AE onset cutoff values were determined at 56% and 252 Torr, respectively, by multivariate and receiver-operating characteristic analyses for predicting mortality within 90 days of AE onset, with C-indices of 0.566 and 0.692, respectively (Supplementary Fig. S1). The post-AE 90-day survival rate was higher in the patients with baseline %FVC ≥ 56% than in those with baseline %FVC < 56% (72.6% vs. 40.7%, respectively; *p* < 0.01; Fig. 3b) and in the patients with P/F ratio at AE onset ≥ 252 Torr than in those with ratios < 252 Torr (82.2% vs. 50.8%, respectively; *p* < 0.01; Fig. 3c).

For each prognostic factor, 1 point was assigned with a P/F ratio at AE onset < 252 Torr and baseline %FVC < 56%, and the patients were categorized into 3 groups based on total point scores as follows: mild (0), moderate (1), and severe (2). The post-AE 90-day cumulative survival rates of mild, moderate, and severe groups were 83.3%, 66.2%, and 22.2%, respectively (Fig. 3d). This model showed significant differences in adjacent survival curves (mild vs. moderate, adjusted *p* = 0.04; moderate vs. severe, adjusted *p* < 0.01). The discrimination performance (C-index) of this model for mortality within 90 days of AE onset was 0.702.

### Prognostic classification model 2

**Figure 4:**
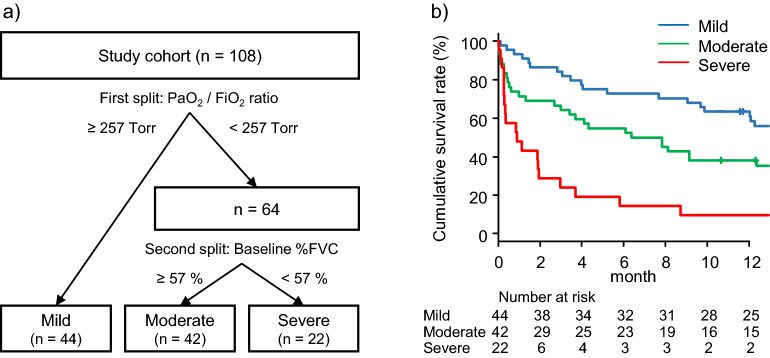
Prognostic classification model 2. (**a**) A decision tree predicting 90-day mortality created by recursive partitioning. The splitting process terminated when the study cohort was divided into 3 groups as follows: mild, moderate, and severe. The variables (cut points) of the optimal split in the first and second split candidates were PaO_2_/FiO_2_ ratio (257 Torr) and baseline % FVC (57%), respectively. (**b**) The post-AE 90-day cumulative survival rates of the patients classified by decision tree: mild group, 84.1% (95% CI 69.5–92.1); moderate group, 64.3% (95% CI 47.9–76.7); and severe group, 24.0% (95% CI 8.8–43.3), with the mild group compared with the moderate group (adjusted *p* = 0.01 by Wilcoxon test) and the moderate group compared with the severe group (adjusted *p* < 0.01 by Wilcoxon test). The discrimination performance (C-index) of this model for post-AE 90-day survival was 0.735 (95% CI 0.639–0.831). *AE* acute exacerbation, *FVC* forced Vital Capacity, *P/F* PaO_2_-to-FiO_2_ ratio.

Figure 4a shows a decision tree predicting 90-day mortality created by recursive partitioning. The splitting process terminated when the study cohort was divided into 3 groups as follows: mild, moderate, and severe. The candidate reports for the first and second splits are shown in Supplementary Tables S2 and S3, respectively. The variables (cutoff points) of the optimal split in the first and second splits were PaO_2_/FiO_2_ ratio (257 Torr) and baseline % FVC (57%), respectively. The post-AE 90-day cumulative survival rates of the mild, moderate, and severe groups were 84.1%, 64.3%, and 24.0%, respectively (Fig. 4b). This model also showed significant differences in adjacent survival curves (mild vs. moderate, adjusted *p* = 0.01; moderate vs. severe, adjusted *p* < 0.01). The discrimination performance (C-index) of this model for mortality within 90 days of AE onset was 0.735.

### Subanalyses on AE relapse

Table 3 shows results of subdistribution hazards analysis of AE relapse. In the univariate analysis, the usual interstitial pneumonia pattern on HRCT (vs. other patterns) and higher P/F ratio at AE onset were associated with AE relapse. In the multivariate analysis, higher P/F ratio at AE onset were independently associated with a higher incidence of AE relapse. Cox proportional hazards analysis with time-dependent covariates demonstrated that AE relapsing (a time-dependent covariate) was significantly associated with a shorter survival after adjustment for the identified prognostic factors (adjusted HR, 6.47; 95% CI 3.03–13.8; *p* < 0.01) (Supplementary Table S4).

**Table 3:** Results of fine-gray subdistribution hazards analysis of AE relapse.

	HR	95% CI	*p* value
Univariate model			
Male (vs. female)	0.21	0.03–1.58	0.13
Smoking, current or ex (vs. never)	2.65	0.65–10.89	0.18
Baseline % FVC^a^, per 1% increase	1.00	0.99–1.01	0.67
Baseline % DL_CO_^a^, per 1% increase	1.03	0.99–1.02	0.70
UIP pattern on HRCT^a^ (vs. other patterns)	0.40	0.17–0.98	0.045
Treatment for IPF prior to AE			
Anti-inflammatory, yes (vs. no)	0.63	0.19–2.08	0.45
Antifibrotic, yes (vs. no)	2.21	0.94–5.17	0.068
Pirfenidone	2.07	0.81–5.27	0.13
Nintedanib	1.99	0.43–9.08	0.38
At AE onset			
Age, years	0.99	0.95–1.03	0.69
PaO_2_/FiO_2_ ratio, per 10 Torr increase	1.06	1.02–1.11	< 0.01
KL-6, per 100 U/mL increase	1.00	0.98–1.03	0.76
SP-D, per 100 ng/mL increase	1.07	0.97–1.18	0.17
First-line therapy for first AE			
CS + IS (vs. CS)	1.23	0.58–2.58	0.59
PMX-DHP, yes	1.40	0.62–3.16	0.42
Multivariate model			
Baseline % FVC^a^, per 1% increase	1.01	0.99–1.03	0.50
Baseline % DL_CO_^a^, per 1% increase	0.98	0.96–1.002	0.07
UIP pattern on HRCT^a^ (vs. other patterns)	0.34	0.11–1.08	0.07
PaO_2_/FiO_2_ ratio at AE onset, per 10 Torr increase	1.10	1.04–1.16	< 0.01

*HR* hazards ratio, *CI* confidence interval, *AE* acute exacerbation, *FVC* forced vital capacity, *DL*_*CO*_ diffusing capacity of the lung carbon monoxide, *UIP* usual interstitial pneumonia, *HRCT* high-resolution computed tomography, *CS* corticosteroids, *IS* immunosuppressant, *PMX-DHP* polymyxin-B direct hemoperfusion, *KL-6* Krebs von den Lungen-6, *SP-D* surfactant protein D.

^a^Within 12 months before first AE.

## Discussion

This is the largest study to identify prognostic factors and develop simple-to-use prognostic classification models in patients with AE-IPF. In this cohort, post-AE 90-day and 1-year survival rates were approximately 60% and 40%, respectively, and 1-year AE relapse rate was approximately 20%. On multivariate analysis, lower baseline %FVC and lower P/F ratio at AE onset were independent prognostic factors for mortality. Relapsing AE was also associated with shorter survival. With both the scoring model based on the identified prognostic factors, baseline %FVC, and P/F ratio at AE onset and the decision tree-based model created by recursive partitioning, patients with AE-IPF were successfully classified into 3 groups according to different prognoses, namely mild, moderate, and severe.

Studies have reported candidate prognostic factors, including %FVC and DL_CO_^8^, P/F ratio^9,13,14^, HRCT patterns^15,16^, Acute Physiology and Chronic Health Evaluation II score^14^, Glasgow prognostic score^9^, and serum biomarkers (e.g., C-reactive protein, Krebs von den Lungen-6)^7,13,14,17^. Among these, P/F ratio at AE onset has been reported to be the most reproducible prognostic factor^10^. Consistent with these reports, the current study demonstrated that a lower P/F ratio at AE onset was an independent prognostic factor. However, prognostic significance of other candidate factors have been inconsistent across studies, probably due to relatively small retrospective studies with statistical limitation and different study designs^10^. In particular, the evidence on baseline %FVC and %DL_CO_—widely described in the literature as prognostic factors for AE-IPF as well as IPF—is based only on a comparative analysis of AE-IPF survivors and nonsurvivors, data that must be validated in a larger cohort^8^. In this context, the current study demonstrated that a lower baseline %FVC was independently associated with mortality among > 100 patients with AE-IPF. These results suggest that assessment of both oxygen status at acute phase (AE onset) and background pulmonary function (e.g., degree of lung fibrosis) is necessary to better predict prognosis for AE-IPF patients, unlike ARDS as a potentially similar condition^11^.

This study was the first to use the GAP model to determine whether it could classify the prognosis for AE-IPF patients, although it was originally a prediction model for prognosis for IPF patients^2^. On the basis of this model, AE-IPF patients were classified into 3 groups; however, this model failed to clearly separate the adjacent survival curves between the GAP I and II groups and between the GAP II and III groups (Fig. 3a; C-index 0.526). Next, a prognostic classification model (model 1) was built on the basis of the prognostic factors identified by multivariate analysis, baseline %FVC, and P/F ratio at AE onset. This simple model clearly separated the adjacent survival curves of the mild, moderate, and severe groups with statistical significance and showed improved discrimination performance (Fig. 3d; C-index 0.702). In addition, a decision-tree-based prognostic classification model (model 2) was created by recursive partitioning. This model, as in model 1, used baseline %FVC and P/F ratio at AE onset from among the multivariables; accordingly, the patients were successfully classified into 3 groups with different prognoses. The discrimination performance of this model was comparable with that of model 1 (Fig. 4b; C-index 0.735). Prospective studies are needed to validate the usefulness of these models.

The 2011 IPF international Guideline made a weak recommendation for corticosteroid therapy for AE-IPF patients, but no evidence of a specific treatment that significantly improves prognoses has yet been established^18^. However, the results of clinical trials suggested that antifibrotic agents, especially nintedanib, may reduce the incidence of AE^19–22^. To improve the prognosis of IPF patients, the concept of preventing AE is clinically relevant. Also, recent advances in multidisciplinary treatment, including immunosuppressive therapy, polymyxin-B direct hemoperfusion, invasive ventilation, and lung transplantation, appear to have gradually reduced the mortality rates^23^. However, while such treatments include potential benefits, they may be associated with an increased risk of adverse events and higher medical costs. Moreover, some patients do not improve despite treatment and may be considered for end-of-life decisions^24^. In this regard, the simple-to-use prognostic classification models proposed herein may help clinicians determine treatment strategies and inform patient/family decision making. These models may also provide useful information when designing future clinical trials to determine inclusion criteria and stratification.

Some patients with IPF experience AE relapse, despite surviving the initial AE; however, the incidence of AE relapse, its risk factors, and its impact on prognosis are not well understood. In the current study, the 1-year AE relapse rate was approximately 20%, with adjustment for mortality as a competing factor, which appears to be higher than the reported annual incidence of first AE^3^. In the present analysis, a high P/F ratio at initial AE onset was an independent risk factor of AE relapse, presumably because patients with a lower P/F ratio die early, whereas those with a higher P/F ratio have an increased probability of surviving long enough to relapse. No other risk factors for AE relapse could be identified. Importantly, multivariate analysis with time-dependent covariates revealed that AE relapse had a significant impact on patient prognosis. Therefore, management and treatment should also be established to reduce AE relapse.

The present study had several limitations. First, the retrospective design renders it vulnerable to several biases. Second, with the goal to build simple and feasible models based on clinical/physiologic factors, the prognostic significance of non-specific blood biomarkers, such as C-reactive protein or HRCT assessment was not examined. Particularly, quantitative assessment of abnormalities on HRCT requires evaluation by a chest radiologist and thus may not be immediately useful in clinical practice. Third, the baseline %FVC used was within 12 months before AE onset, so differences in timing of the measurement may have influenced the results. In clinical practice, however, AE development is unpredictable, and it is also not easy to measure the %FVC at AE onset, so it may be relatively feasible to use %FVC measured within 12 months before AE onset. Fourth, these models are not applicable in cases in which %FVC was not measured before AE onset. In such cases, prognostic classification should be based solely on P/F ratio at AE onset. Fifth, the different treatment regimens may have affected the outcomes in the study population. Finally, few patients had been introduced to an antifibrotic agent, especially nintedanib, prior to AE onset in this study. This may be because this study partially included patients who developed AE in the period before the widespread use of antifibrotic agents, but it is also possible that after the period of widespread use of antifibrotic agents, the introduction of antifibrotic agents may have reduced the risk of AE development, resulting in a relatively large number of patients with AE who were not taking antifibrotic agents. Therefore, the results of this study should be validated in patients in the current era, where antifibrotic agents are widely used.

In conclusion, this large cohort study demonstrated that lower baseline %FVC and lower P/F ratio at AE onset were independent prognostic factors for mortality in patients with AE-IPF. These simple-to-use prognostic classification models based on baseline %FVC and P/F ratio at AE onset may be useful for predicting prognosis in AE-IPF patients. These results will help guide clinicians to determine a therapeutic strategy, inform patients/families in decision making, and help design future studies on AE-IPF.

## Supplementary Information

Supplementary Information

## Data Availability

The data that support the findings of this study are available from the Hamamatsu University School of Medicine, but restrictions apply to the availability of these data, which were used under license for the present study, and so are not publicly available. Data are, however, available from the authors upon reasonable request and with permission of the Hamamatsu University School of Medicine.

## References

[1] Raghu G (2018). Diagnosis of idiopathic pulmonary fibrosis. An official ATS/ERS/JRS/ALAT clinical practice guideline. Am. J. Respir. Crit. Care Med..

[2] Ley B, Collard HR, King TE (2011). Clinical course and prediction of survival in idiopathic pulmonary fibrosis. Am. J. Respir. Crit. Care Med..

[3] Collard HR (2016). Acute exacerbation of idiopathic pulmonary fibrosis. An international working group report. Am. J. Respir. Crit. Care Med..

[4] Collard HR (2007). Acute exacerbations of idiopathic pulmonary fibrosis. Am. J. Respir. Crit. Care Med..

[5] Kreuter M, Koegler H, Trampisch M, Geier S, Richeldi L (2019). Differing severities of acute exacerbations of idiopathic pulmonary fibrosis (IPF): insights from the INPULSIS® trials. Respir. Res..

[6] Kondoh Y (2010). Risk factors of acute exacerbation of idiopathic pulmonary fibrosis. Sarcoidosis Vasc. Diffuse Lung Dis..

[7] Song JW, Hong SB, Lim CM, Koh Y, Kim DS (2011). Acute exacerbation of idiopathic pulmonary fibrosis: incidence, risk factors and outcome. Eur. Respir. J..

[8] Simon-Blancal V (2012). Acute exacerbation of idiopathic pulmonary fibrosis: outcome and prognostic factors. Respiration.

[9] Kang HS, Cho KW, Kwon SS, Kim YH (2018). Prognostic significance of Glasgow prognostic score in patients with acute exacerbation of idiopathic pulmonary fibrosis. Respirology.

[10] Kamiya H, Panlaqui OM (2020). Systematic review and meta-analysis of prognostic factors of acute exacerbation of idiopathic pulmonary fibrosis. BMJ Open.

[11] Ranieri VM (2012). Acute respiratory distress syndrome: the Berlin definition. JAMA.

[12] Ley B (2012). A multidimensional index and staging system for idiopathic pulmonary fibrosis. Ann. Intern. Med..

[13] Kishaba T, Tamaki H, Shimaoka Y, Fukuyama H, Yamashiro S (2014). Staging of acute exacerbation in patients with idiopathic pulmonary fibrosis. Lung.

[14] Kawamura K, Ichikado K, Yasuda Y, Anan K, Suga M (2017). Azithromycin for idiopathic acute exacerbation of idiopathic pulmonary fibrosis: a retrospective single-center study. BMC Pulm. Med..

[15] Akira M, Kozuka T, Yamamoto S, Sakatani M (2008). Computed tomography findings in acute exacerbation of idiopathic pulmonary fibrosis. Am. J. Respir. Crit. Care Med..

[16] Fujimoto K (2012). Acute exacerbation of idiopathic pulmonary fibrosis: high-resolution CT scores predict mortality. Eur. Radiol..

[17] Suzuki A (2018). Prognostic evaluation by oxygenation with positive end-expiratory pressure in acute exacerbation of idiopathic pulmonary fibrosis: A retrospective cohort study. Clin. Respir. J..

[18] Raghu G (2011). An official ATS/ERS/JRS/ALAT statement: idiopathic pulmonary fibrosis: evidence-based guidelines for diagnosis and management. Am. J. Respir. Crit. Care Med..

[19] Azuma A (2005). Double-blind, placebo-controlled trial of pirfenidone in patients with idiopathic pulmonary fibrosis. Am. J. Respir. Crit. Care Med..

[20] Richeldi L (2011). Efficacy of a tyrosine kinase inhibitor in idiopathic pulmonary fibrosis. N. Engl. J. Med..

[21] Richeldi L (2014). Efficacy and safety of nintedanib in idiopathic pulmonary fibrosis. N. Engl. J. Med..

[22] Richeldi L (2016). Nintedanib in patients with idiopathic pulmonary fibrosis: combined evidence from the TOMORROW and INPULSIS(®) trials. Respir. Med..

[23] Kondoh Y, Cottin V, Brown KK (2017). Recent lessons learned in the management of acute exacerbation of idiopathic pulmonary fibrosis. Eur. Respir. Rev..

[24] Akiyama N (2020). Palliative care for idiopathic pulmonary fibrosis patients: pulmonary physicians' view. J. Pain Symptom Manag..

