# Supplementary material for: Prognostic classification in acute exacerbation of idiopathic pulmonary fibrosis: a multicentre retrospective cohort study
Source: Sci Rep. 2021 Apr 27;11:9120. doi: 10.1038/s41598-021-88718-2 (PMC8079397; doi:10.1038/s41598-021-88718-2)
Supplement: Supplementary file 1 — Supplementary Information [file 41598_2021_88718_MOESM1_ESM.pdf]

Supplementary Information

Title: Prognostic classification in acute exacerbation of idiopathic pulmonary fibrosis: a multicentre retrospective cohort study

Authors' full names:

Takahito Suzuki, M.D.[1], Hironao Hozumi, M.D., Ph.D.[1*], Koichi Miyashita, M.D.[1], Masato Kono, M.D., Ph.D.[2], Yuzo Suzuki, M.D., Ph.D.[1], Masato Karayama, M.D., Ph.D.[1], Kazuki Furuhashi, M.D., Ph.D.[1], Hirotsugu Hasegawa, M.D., Ph.D.[3], Tomoyuki Fujisawa, M.D., Ph.D.[1], Noriyuki Enomoto, M.D., Ph.D.[1], Yutaro Nakamura, M.D., Ph.D.[1], Naoki Inui, M.D., Ph.D.[1, 4], Koshi Yokomura, M.D., Ph.D.[3], Hidenori Nakamura, M.D., Ph.D.[2], Takafumi Suda, M.D., Ph.D.[1]

Authors' affiliation(s):

[1] Second Division, Department of Internal Medicine, Hamamatsu University School of Medicine, Hamamatsu, Japan

[2] Department of Respiratory Medicine, Seirei Hamamatsu General Hospital, Hamamatsu, Japan

[3] Department of Respiratory Medicine, Seirei Mikatahara General Hospital, Hamamatsu, Japan

[4] Department of Clinical Pharmacology and Therapeutics, Hamamatsu University School of Medicine, 1-20-1 Handayama Higashiku, Hamamatsu 431-3192, Japan

Supplementary Table S1. Results of the Multivariate Cox Hazards Analysis Adjusted for AE Treatment

	**HR**	**95% CI**	***P*-value**
Age at AE onset, years	1.02	0.98–1.07	0.35
Male (vs. female)	0.73	0.29–1.88	0.52
Baseline % FVC ‡, per 1% increase	0.97	0.95–0.99	<0.01
Baseline % DL_{CO} ‡, per 1% increase	1.01	0.99–1.03	0.13
Anti-inflammatory treatment prior to AE, yes (vs. no)	1.86	0.85–4.05	0.12
PaO_2 / FiO_2 ratio at AE onset, per 10 Torr increase	0.89	0.84–0.95	<0.01
First-line therapy for first AE			
CS + IS (vs. CS)	1.24	0.67–2.30	0.50
PMX-DHP, yes	0.79	0.40–1.56	0.49

‡ within 12 months before first AE

HR, hazards ratio; CI, confidence interval; AE, acute exacerbation; FVC, forced vital capacity; DL_{CO}, diffusing capacity of the lung carbon monoxide, CS, corticosteroids; IS, immunosuppressant; PMX-DHP, polymyxin-B direct hemoperfusion

Supplementary Table S2. Candidate Report for the First Split

	**Candidate G^2**	**Log Worth**	**Cutoff Point**
Male (vs. female)	1.147	0.547	Male
Smoking, current or ex (vs. never)	1.415	0.630	Current or ex
Baseline % FVC, % ‡	8.462	1.187	57
Baseline % DL_{CO}, % ‡	5.662	0.623	44
Baseline GAP stage	1.361	0.348	II
UIP pattern on HRCT ‡ (vs. other patterns)	0.081	0.110	UIP
Treatment for IPF prior to AE			
Anti-inflammatory, yes (vs. no)	6.567	1.983	Yes
Anti-fibrotic, yes (vs. no)	0.973	0.490	Yes
At AE onset			
Age, years	1.639	0.084	64
PaO_2/FiO_2 ratio, Torr	11.801	2.020	257*
KL-6, U/mL	7.841	0.855	634
SP-D, ng/mL	6.193	0.546	510
First-line therapy for first AE			
CS + IS (vs. CS)	0.0003	0.007	Yes
PMX-DHP, yes	0.187	0.177	Yes

*Optimal split

‡Within 12 months before the first AE onset

Candidate G^2, Likelihood ratio chi-square for the best split; LogWorth, the LogWorth statistics; AE, acute exacerbation; FVC, forced vital capacity; DL_{CO}, diffusing capacity of the lung carbon monoxide; UIP, usual interstitial pneumonia; HRCT, high-resolution computed tomography; CS, corticosteroids; IS, immunosuppressant; PMX-DHP, polymyxin-B direct hemoperfusion; KL-6, Krebs von den Lungen-6; SP-D, surfactant protein D

Supplementary Table S3. Candidate Report for the Second Split

	Candidate G^2	Log Worth	Cutoff Point
Male (vs. female)	2.585	0.967	Male
Smoking, current or ex (vs. never)	3.644	1.250	Current or ex
Baseline % FVC, % ‡	9.224	1.460	57*
Baseline % DL_{CO}, % ‡	4.883	0.592	37
Baseline GAP stage	0.465	0.126	II
UIP pattern on HRCT ‡ (vs. other patterns)	2.510	0.946	UIP
Treatment for IPF prior to AE			
Anti-inflammatory, yes (vs. no)	3.320	1.165	Yes
Anti-fibrotic, yes (vs. no)	0.468	0.306	Yes
At AE onset			
Age, years	1.501	0.037	64
PaO_2 / FiO_2 ratio, Torr	2.976	0.244	157
KL-6, U/mL	3.320	0.211	917
SP-D, ng/mL	8.366	1.186	209
First-line therapy for first AE			
CS + IS (vs. CS)	0.017	0.047	Yes
PMX-DHP, yes	0.303	0.235	Yes

*Optimal split

‡Within 12 months before the first AE onset

Candidate G^2, Likelihood ratio chi-square for the best split; Log Worth, the LogWorth statistics, defined as −log10(p-value); AE, acute exacerbation; FVC, forced vital capacity; DL_{CO}, diffusing capacity of the lung carbon monoxide; UIP, usual interstitial pneumonia; HRCT, high-resolution computed tomography; CS, corticosteroids; IS, immunosuppressant; PMX-DHP, polymyxin-B direct hemoperfusion; KL-6, Krebs von den Lungen-6; SP-D, surfactant protein D

Supplementary Table S4. Multivariate Cox Proportional Hazards Regression Analysis with Time-Dependent Covariates of Mortality

	**HR**	**95% CI**	***P*-value**
AE relapse*	6.47	3.03–13.8	<0.01
Age at AE onset, years	1.03	0.99–1.07	0.15
Male (vs. female)	0.49	0.18–1.31	0.15
Baseline % FVC ‡, per 1% increase	0.97	0.95–0.99	0.01
Baseline % DL_{CO} ‡, per 1% increase	1.01	0.99–1.03	0.11
Anti-inflammatory treatment prior to AE, yes (vs. no)	2.01	0.93–4.34	0.08
PaO_2 / FiO_2 ratio at AE onset, per 10 Torr increase	0.89	0.84–0.95	<0.01

*Time-dependent covariate

‡Within 12 months before first AE

HR, hazards ratio; CI, confidence interval; AE, acute exacerbation; FVC, forced vital capacity; DL_{CO}, diffusing capacity of the lung carbon monoxide

Supplementary Figure S1. Receiver operating characteristic curves.

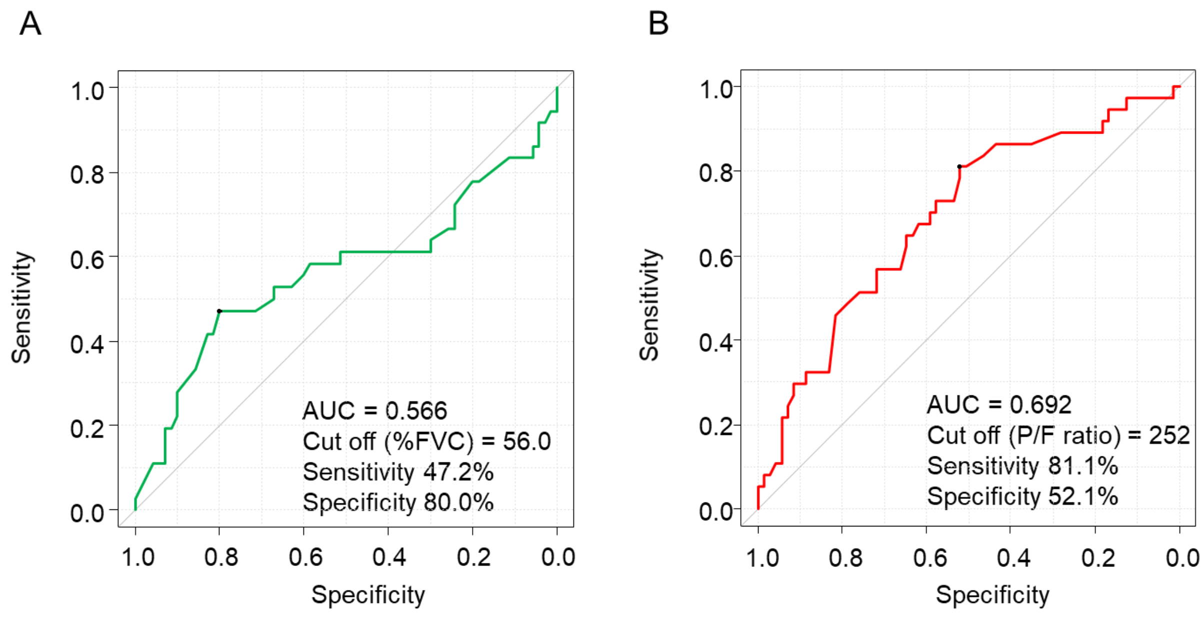

A) ROC curve of %FVC for predicting death within 90 days after AE onset. The AUC was 0.566 (95% CI, 0.435–0.695). Using 56.0 as the cutoff level of %FVC, the sensitivity and specificity were 47.2% and 80.0%, respectively.

B) ROC curve of P/F ratio for predicting death within 90 days after AE onset. The AUC was 0.692 (95% CI, 0.586–0.798). Using 252 as the cutoff level of P/F ratio, the sensitivity and specificity were 81.1% and 52.1%, respectively.

ROC, receiver operating characteristic; CI, confidence interval; FVC, forced vital capacity; P/F, PaO_2 to FiO_2 ratio; AE, acute exacerbation; AUC, area under the curve
